# Deep Learning Spinal Cord Segmentation Based on B0 Reference for Diffusion Tensor Imaging Analysis in Cervical Spondylotic Myelopathy †

**DOI:** 10.3390/bioengineering12070709

**Published:** 2025-06-28

**Authors:** Shuoheng Yang, Ningbo Fei, Junpeng Li, Guangsheng Li, Yong Hu

**Affiliations:** 1 Spinal Division, Orthopedic and Traumatology Center, The Affiliated Hospital of Guangdong Medical University, Zhanjiang 524002, China; shuoheng@connect.hku.hk (S.Y.); junpengli233@gdmu.edu.cn (J.L.); liguangsheng@gdmu.edu.cn (G.L.); 2 Department of Orthopaedics and Traumatology, The University of Hong Kong, Hong Kong SAR, China; u3007507@connect.hku.hk

**Keywords:** diffusion tensor imaging, cervical spondylotic myelopathy, medical image segmentation, deep learning

## Abstract

Diffusion Tensor Imaging (DTI) is a crucial imaging technique for accurately assessing pathological changes in Cervical Spondylotic Myelopathy (CSM). However, the segmentation of spinal cord DTI images primarily relies on manual methods, which are labor-intensive and heavily dependent on the subjective experience of clinicians, and existing research on DTI automatic segmentation cannot fully satisfy clinical requirements. Thus, this poses significant challenges for DTI-assisted diagnostic decision-making. This study aimed to deliver AI-driven segmentation for spinal cord DTI. To achieve this goal, a comparison experiment of candidate input features was conducted, with the preliminary results confirming the effectiveness of applying a diffusion-free image (B0 image) for DTI segmentation. Furthermore, a deep-learning-based model, named SCS-Net (Spinal Cord Segmentation Network), was proposed accordingly. The model applies a classical U-shaped architecture with a lightweight feature extraction module, which can effectively alleviate the training data scarcity problem. The proposed method supports eight-region spinal cord segmentation, i.e., the lateral, dorsal, ventral, and gray matter areas on the left and right sides. To evaluate this method, 89 CSM patients from a single center were collected. The model demonstrated satisfactory accuracy for both general segmentation metrics (precision, recall, and Dice coefficient) and a DTI-specific feature index. In particular, the proposed model’s error rate for the DTI-specific feature index was evaluated as 5.32%, 10.14%, 7.37%, and 5.70% on the left side, and 4.60%, 9.60%, 8.74%, and 6.27% on the right side of the spinal cord, respectively, affirming the model’s consistent performance for radiological rationality. In conclusion, the proposed AI-driven segmentation model significantly reduces the dependence on DTI manual interpretation, providing a feasible solution that can improve potential diagnostic outcomes for patients.

## 1. Introduction

Diffusion Tensor Imaging (DTI) is a neuroimaging technique that allows precise evaluation of pathological conditions for every corticospinal tract in the cervical cord and accurate diagnosis of cervical spondylotic myelopathy (CSM) [1,2,3,4]. DTI features such as FA (Fractional Anisotropy) values can demonstrate the demyelination of nerve fiber bundles, which gives insights of pathological state changes in the spinal cord in CSM [5,6]. Since the spinal cord is comprised of various tracts, including ascending pathways, descending pathways, and gray matter, accurate evaluation of tract-specific conditions is critical for the diagnosis of CSM and for guiding appropriate clinical decision-making. Consequently, some studies have suggested that the identification of distinct regions of interest (ROIs) in spinal cord DTI analysis is essential [7,8,9].

Currently, the acquisition of ROIs requires that clinicians manually draw the spinal cord as detailed anatomical regions, a process that is both time-consuming and labor-intensive [9]. Moreover, the manual drawing process heavily relies on the clinician’s expertise in interpreting DTI data, leading to subjectivity and variability between clinicians, which can potentially compromise the reliability of DTI-assisted clinical applications. To address these challenges, automatic segmentation strategies are considered a feasible solution.

In recent years, deep-learning-based automated image segmentation [10] has made significant progress in many medical application fields [11,12]. In the field of medical image segmentation, U-shape structure-based models have been widely applied because of their architectural flexibility and stable performance under a limited amount of training data [13,14,15]. More recently, Vision Transformers (ViTs) [16] models have been proposed as an advanced alternative to Convolutional Neural Network (CNN)-based segmentation strategies, achieving notable advancements across various tasks [17,18,19,20]. These works have promoted extensions for applying Deep Learning technology in automatic DTI segmentation.

In the field of automatic DTI segmentation, Richu et al. [9] provided comparison segmentation results for DTI using SVM and a deep learning (DL) model, but the results showed that deep learning still needs to be refined to produce a superior performance. Ningbo et al. [21] provided detailed segmentation results of CSM patients’ DTI data solely based on DL technology, demonstrating the feasibility of deep-learning-based segmentation for cervical spinal cord DTI.

However, existing studies on the clinical applications of DTI segmentation are constrained by three major factors.

- First, the selection of appropriate input feature images for DTI segmentation remains unclear. DTI typically includes several forms of diffusive feature images, the most commonly accepted forms are the FA value image. On the other hand, diffusion-free images (B0 image), which are collected for DTI registration, are a promising alternative input for DTI ROI acquisition, this is because of the clear anatomical information contained and the shared ROI localization with DTI images. However, the feasibility of these kinds of candidate inputs for DTI segmentation remains unclear.
- Second, the scarcity of DTI data (including B0 image) constrains the models from achieving multi-class segmentation. This limitation poses a challenge, as detailed anatomical-level segmentation of the spinal cord is necessary in clinical usage.
- Third, beyond general segmentation performance, ensuring radiological consistency between the predicted segments and the ground truth is required. This is because errors in the predicted ROI segments can impact subsequent ROI-based diffusive feature analyses. However, existing methods have not adequately demonstrated such consistency, which may affect the clinical reliability of their predicted outcomes.

The objective of this study is to promote the clinical application of spinal cord DTI segmentation in CSM. By developing a deep-learning-based segmentation method, this study aims to make contributions in the following key aspects:

- First, preliminary candidate input feature selection was performed through a comparative experiment between B0 and FA images. The results confirmed that using B0 images yielded better segmentation performance. Because of the shared ROI localization of B0 and DTI image, this preliminary finding motivated a practical strategy applying the projection of B0-based segmentation onto FA images for DTI segmentation.
- Second, to address data scarcity challenges, a DL-based novel network, SCS-Net (Spinal Cord Segmentation Network) was proposed. SCS-Net incorporates a customized lightweight feature extraction block within a classical U-shaped architecture, effectively mitigating the data scarcity for multi-class segmentation by fully leveraging the model structure, while reducing the inter-structure complexity. The results showed that SCS-Net achieved state-of-the-art performance compared to existing methods.
- Third, to evaluate the model’s radiological consistency, a DTI-specific feature evaluation index was applied in the model evaluation. Evaluation based on this index demonstrated the proposed network’s radiological consistency, validating its reliability and applicability in clinical usage.

Additionally, the proposed network was further encapsulated into a streamlined pipeline [22]. To the best of our knowledge, this is the first and only pipeline specifically designed for clinical-level spinal cord DTI anatomical segmentation [23]. The delivered methods and pipeline reduce the workload of clinicians in acquiring DTI ROIs by providing a uniform segmentation result, which will enhance the convenience of spinal cord DTI-based clinical decision-making.

This work is a revised and expanded version of a conference proceeding paper [22].

## 2. Materials and Methods

### 2.1. Dataset and Preprocessing

The dataset for this study was constructed using data from patients diagnosed with cervical spondylotic myelopathy (CSM). A total of 89 symptomatic subjects were recruited, all of whom underwent clinical evaluation using the Japanese Orthopedic Association (JOA) scoring system, with scores ranging from 2 to 15 and a mean value of 10.4 ± 2.4. The ages of the participants ranged from 26 to 98 years, with a mean age of 65.7 ± 13.9 years. The cohort consisted of 61 males and 28 females.

The inclusion criteria required a clinical diagnosis of CSM without any history of spinal surgery. The exclusion criteria ruled out individuals with a prior diagnosis of other neurological disorders or a history of neurological trauma.

Typically, each patient’s DTI (including B0 image) comprised 12 slices, resulting in a total collection of 1059 slices after excluding images that were challenging to segment manually. Regarding the original DTI acquisition process, this was conducted using a Philips 3T Achieva scanner(Philips Medical System, Best, Netherland), and a Single-shot Echo Planar Imaging (EPI) sequence was used with EPI factor = 35. During DTI collection, one b-shell setting was employed, with b values equal to 600 s/mm^2^ for 15 nonlinear and noncoplanar directions, and a number of excitations (NEX) = 3. Table 1 outlines other parameter settings related to the DTI equipment used for generating the original data files. To generate an input source compatible with computer vision technologies, the DTI slices were processed using the Spinal Cord Toolbox (Version 2.3). Subsequently, FA-value-based images were derived from the B0 images through a specific computational process. In the subsequent experiments, both B0 and FA images were utilized as input sources, whereas only FA images were used for the final demonstration of the segmentation results. All original data were saved as ‘.png’ format, with image dimensions fixed at [128 × 128].

**Figure 1:**
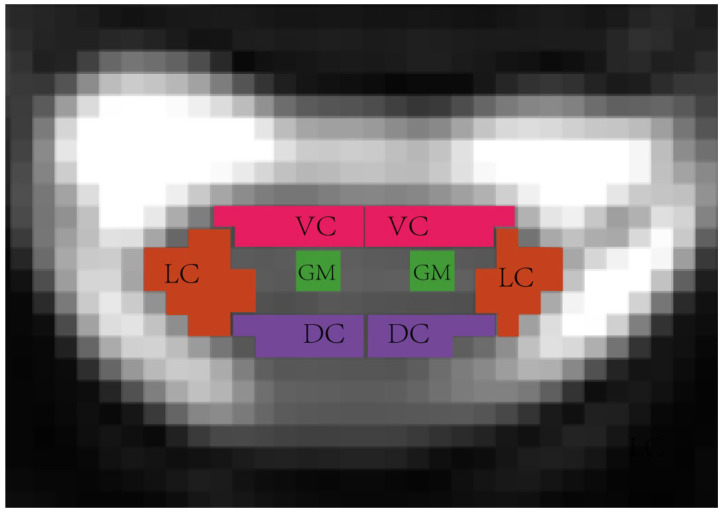
Spinal cord anatomical-level ROI visualization, in the segmentation experiment, the ROI definitions distinguished between the anatomical structures on the left and right sides, including the left lateral column (L_LC), left dorsal column (L_DC), left ventral column (L_VC), left gray matter(L_GM), right lateral column (R_LC), right dorsal column (R_DC), right ventral column (R_VC), and right gray matter (R_GM).

The labeling process of the original DTI slices was through manual annotation, and several experienced researchers contributed by manually annotating and managing the original images to achieve a detailed ROI drawing of the spinal cord. Based on the knowledge of spinal cord anatomy, a total of eight distinct areas were identified, which will assist medical staff in assessing the compression status of the spinal cord during diagnosis. The eight identified regions included the lateral column (LC), dorsal column (DC), ventral column (VC), and gray matter (GM) on both the left and right sides of the spinal cord, and Figure 1 demonstrates a visualization sample of the identified anatomical regions. Notably, in this segmentation experiment, the ROI definitions distinguished between the anatomical structures on the left and right sides of the spinal cord. As a result, a total of nine ROI classes (including the background) were identified.

### 2.2. Model Training Setting

Regarding the dataset splitting strategy, all slices from the patients were initially combined into a single cluster. The cluster was then divided into three parts: a training set (81%), a validation set (9%), and a testing set (10%), which is same as with the general standard general deep learning task. This splitting process was conducted using random sampling, to ensure a balanced distribution of all slices across the datasets.

### 2.3. Model Structure

As mentioned earlier, U-shaped structure-based models are among the most widely accepted architectures for medical image segmentation tasks. Compared to general semantic segmentation models, U-shaped models are characterized by their low parameter count and strong fitting capability, making them particularly well suited for scenarios with limited training data [13,14]. In this study, we extended these advantages by designing a U-shaped model named SCS-Net, tailored specifically for spinal cord segmentation.

In addition, to ensure effective multi-segmentation performance on a limited dataset, a lightweight feature extraction module was developed by simply integrating residual bottleneck blocks [24]. These blocks have been demonstrated to possess a parameter-efficient structure and can effectively address training instability, thereby enhancing the feature extraction module’s potential for competitive representational power without requiring additional parameters [25].

**Figure 2:**
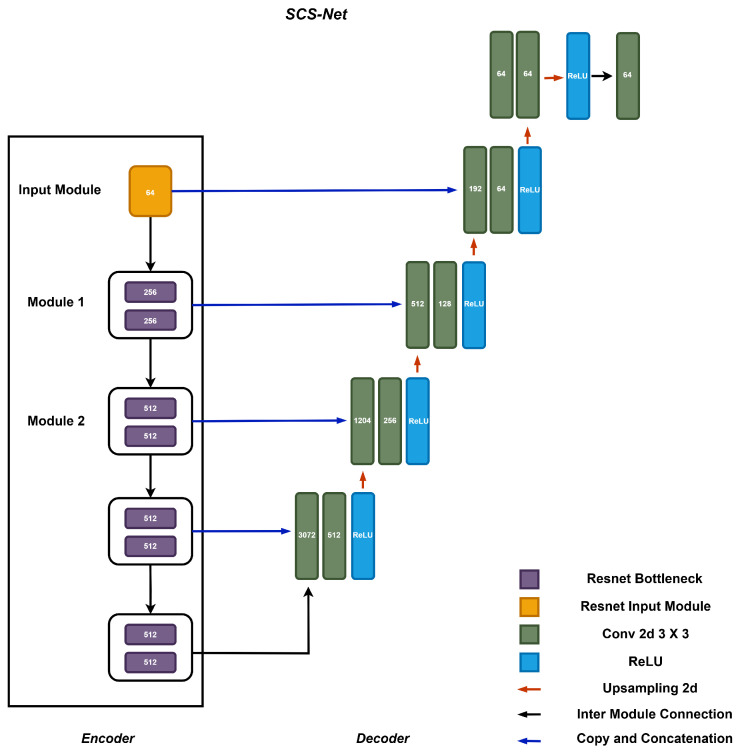
The structure of the proposed segmentation model SCS-Net.

Figure 2 illustrates the detailed architecture of SCS-Net, which adopts a classical U-shaped architecture based on an encoder–decoder design. The encoder of SCS-Net comprises five stacked modules. It begins with an input module that transforms the original input image into a feature map. Subsequently, four stacked feature extraction modules are employed to progressively downsample the feature maps generated at the previous level. This hierarchical structure facilitates the extraction of multi-scale contextual features, ranging from fine-grained to coarse-grained (highly abstract) representations.

The decoder of SCS-Net mirrors the encoder’s structure and consists of an equal number of decoding modules, forming an upsampling path. These modules are responsible for interpreting the encoded features and reconstructing them into meaningful segmentation outputs. Notably, the architecture incorporates cascaded operations within the encoder–decoder design, where feature maps from the encoder are directly concatenated with their corresponding decoding modules via skip connections. This design is widely recognized for enhancing the utilization of enriched spatial information during the decoding process, which is essential for achieving precise segment localization [15,26].

**Figure 3:**
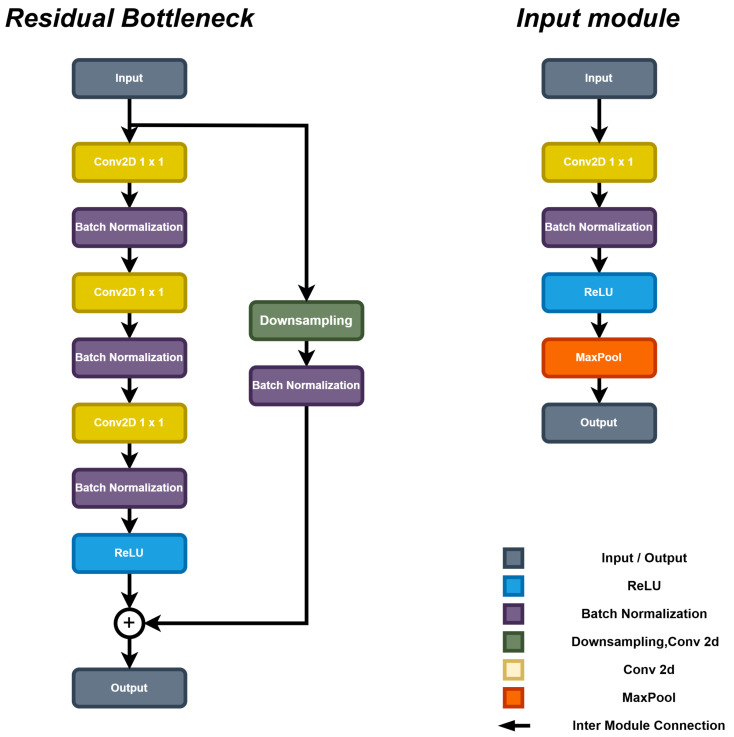
The structure of the feature extraction block of SCS-Net.

The feature extraction module is designed using two residual bottleneck blocks to improve computational efficiency [27]. As illustrated in Figure 3, the core structure of the bottleneck block consists of a group of convolutional layers, integrated with Batch Normalization and ReLU activation functions. This design effectively extracts features, while reducing computational cost through dimensionality reduction (achieved via 1 × 1 convolution operations). Furthermore, a residual connection is incorporated within the bottleneck block to ensure efficient gradient flow during the training process, therefore enhancing the potential model performance [28].

To ensure comparable results, we reproduced several CNN-based segmentation models, specifically a U-Net with a VGG19 [29] backbone (UNet-VG19), a U-Net with a ResNet50 backbone (Unet-RS50) [30], and the U-Net with a VGG16 backbone proposed by Ningbo et al. [21] (referred to as Unet-N). Moreover, a Transformer-based model, Trans-Unet [17], was also reproduced. The performance comparison between CNN-based models with Trans-Unet helped to investigate the effect of model complexity under the current dataset.

### 2.4. Model Training Settings

In this study, the proposed model was trained in a GPU-accelerated environment to improve training efficiency. For the hardware configuration, an Nvidia GeForce 3080Ti GPU (Nvida, Santa Clara, CA, USA) was utilized to accelerate the deep learning training process. The system hardware also included an Intel i5-11400F CPU (Intel, Santa Clara, CA, USA) and 32 GB of DDR4 RAM (ADATA, Taiwan, China). Regarding the software settings, the training was conducted on Windows 10 using PyTorch version 1.10 with GPU support, along with the appropriate CUDA and cuDNN versions, which were essential for deploying the deep learning framework.

In terms of training parameters, the model was trained over 500 epochs using the Adam optimizer. The initial learning rate was set to 1 × 10^−4^, and a learning rate decay strategy was applied to enhance the convergence speed of the model. Table 2 provides a detailed summary of the training parameters employed in this study.

**Figure A1:**
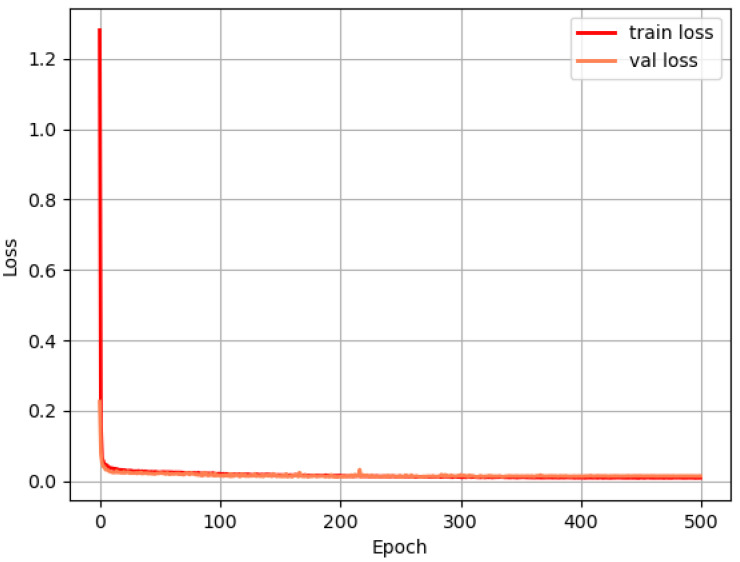
The loss of SCS-Net during the training process.

One crucial hyperparameter in the training process is the loss function. In this study, the focal loss function was selected due to its widespread use in segmentation tasks and its effectiveness in mitigating the impact of sample imbalance across different segmentation classes. The loss of the training process and corresponding explanation are demonstrated in Figure A1.

Additionally, the training procedure was slightly modified by experimentally assigning a weight of 3 to each ROI class (excluding the background), while the background class was assigned a weight of 1. This adjustment encouraged the model to focus more on accurately classifying the critical ROI classes, rather than the background during the training process. The definition of the focal loss function is provided in Equation (1). In all following equations, $g_{n}\left(i\right)$ and $p_{n}\left(i\right)$ denote the ground truth and prediction result of the i-th ROI class in the n-th pixel in input image, respectively, *N* represents the total number of pixels.

(1) $$FocalLoss=-\frac{1}{N}\sum_{i=1}^{I}\sum_{n=1}^{N}g_{n}\left(i\right){\left(1-p_{n}\left(i\right)\right)}^{2}\log\left(p_{n}\left(i\right)\right)$$

### 2.5. Evaluation Method

In order to obtain a comprehensive performance record, both the segmentation evaluation index in the general segmentation task and the specific DTI indicator were selected, and each of these indicators was independently reported for every ROI of spinal cord. For the general segmentation task, precision and recall were selected. To further determine the overall performance of the model among the different evaluation indexes, the Dice coefficient was derived from the above index. The formulation of these indexes is demonstrated below:

(2) $$TP\left(i\right)=\sum_{n=1}^{N}p_{n}\left(i\right)g_{n}\left(i\right)$$

(3) $$FN\left(i\right)=\sum_{n=1}^{N}\left(1-p_{n}\left(i\right)\right)g_{n}\left(i\right)$$

(4) $$FP\left(i\right)=\sum_{n=1}^{N}p_{n}\left(i\right)\left(1-g_{n}\left(i\right)\right)$$

(5) $$Precision\left(i\right)=\frac{TP(i)}{TP(i)+FP(i)}$$

(6) $$Recall\left(i\right)=\frac{TP(i)}{TP(i)+FN(i)}$$

(7) $$Dice\left(i\right)=\frac{2*TP(i)}{2*TP(i)+FN(i)+FP(i)}$$

where TP(i), FN(i), and FP(i) refer to rate of true positives, rate of false negatives, and rate of false positives for the classes. For specific indicators in DTI, a FA value indicator was selected, to provide DTI-specific evaluation. Because the FA values of each part of the spinal cord are different, missed correct pixels or wrongly segmented pixels would cause an FA error with the ground truth, and since the border of each ROI is blurred, the FA-related indicator helped to reveal imaging rationality in the segmentation model. Based on the above principle, we employed the Mean FA Error of each ROI class as the evaluation indicator, which helped to evaluate the segmentation model’s imaging rationality. The formulation of this indicator is given in Equation (8), where C denotes the total number of pixels in i-th ROI class.

(8) $$Mean_{-}FA_{-}ERROR\left(i\right)=\frac{1}{C}\sum_{c}^{C}\frac{\left|FA_{\mathrm{merasure}\;}-FA_{\mathrm{label}\;}\right|}{FA_{\mathrm{label}\;}}$$

### 2.6. Pipeline Design

**Figure 4:**
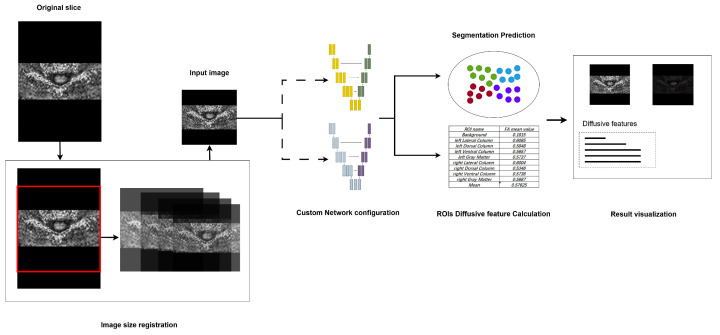
The design of the proposed segmentation pipeline, major steps including image registration, segmentation model configuration, and result visualization.

Regarding the pipeline, a command-line-based operational mechanism was designed to enable rapid segmentation on standard equipment. The standard workflow of the pipeline is illustrated in Figure 4. To enhance accessibility, the workflow incorporated the following three primary steps, streamlining the process for medical professionals and enabling them to utilize advanced segmentation technology, without requiring extensive technical expertise.

First, as the segmentation model requires a fixed-size input, the pipeline incorporates preprocessing steps to standardize images, accommodating those captured under varying devices and environmental conditions. Specifically, images with different dimensions are resized to match the input dimensions through central cropping and rescaling. This method preserves the aspect ratio as closely as possible relative to the original training images. Second, in the network model configuration phase, the pipeline provides flexibility for clinical researchers by allowing them to select either pre-trained models or customized models. This feature extends the pipeline’s applicability to support models trained on other specific datasets, thereby enhancing its adaptability. Lastly, during the segmentation result visualization phase, the pipeline provides a clear and interpretable representation of segmentation outputs. Distinct colors are assigned to different segmented ROIs, alongside the original input images, to improve visual clarity. Furthermore, the mean FA values for each segmented region are displayed during this phase, offering clinicians valuable insights for subsequent analyses.

## 3. Results

### 3.1. Different Model Structure and Input Source

**Figure 5:**
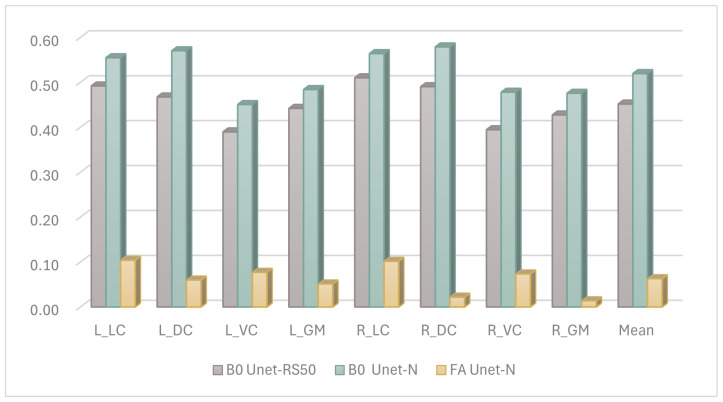
The Dice performance of the different input features for segmentation, ROIs including left lateral column (L_LC), left dorsal column (L_DC), left ventral column (L_VC), left gray matter (L_GM), right lateral column (R_LC), right dorsal column (R_DC), right ventral column (R_VC), right gray matter (R_GM), and Mean result.

Figure 5 illustrates the Dice coefficient results of the U-net model with various encoder backbones and input data modalities. Compared to the U-net model with a ResNet-50 encoder, the Unet-N exhibited superior performance across each ROI class of the spinal cord, achieving the highest performance on the right dorsal column, with a Dice score exceeding 0.55.

Additionally, there was a significant performance disparity observed with different input sources for the segmentation model. Models utilizing images constructed from FA values as the input source achieved performance scores of less than 0.1 on most ROI classes. This was inferred to be due to the FA-value-constructed images appearing brighter, whereas the B0-value-constructed images provided a more recognizable view of the boundaries between ROI classes of the annotated spinal cord. This explanation is consistent with the experience of the clinical staff involved in the manual annotation process.

**Figure 6:**
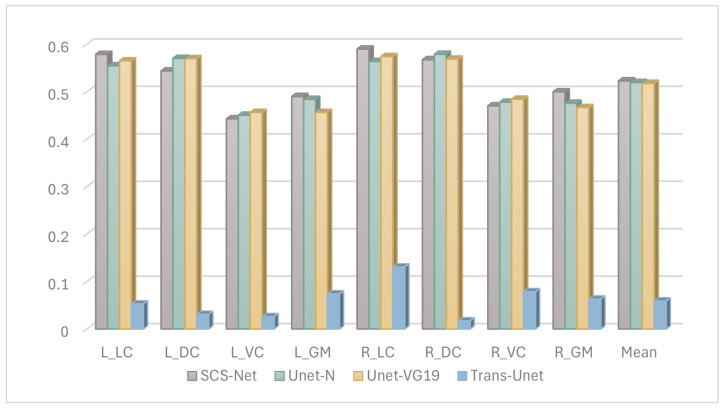
The Dice results of segmentation model performance under DTI B0 images.

Based on the above experience, we further fine-tuned the model structure with B0 images as input, we tested more selections of the model structure, and finally SCS-Net was confirmed as having the best performance, and the results of the compared models are demonstrated in Figure 6.

**Figure 7:**
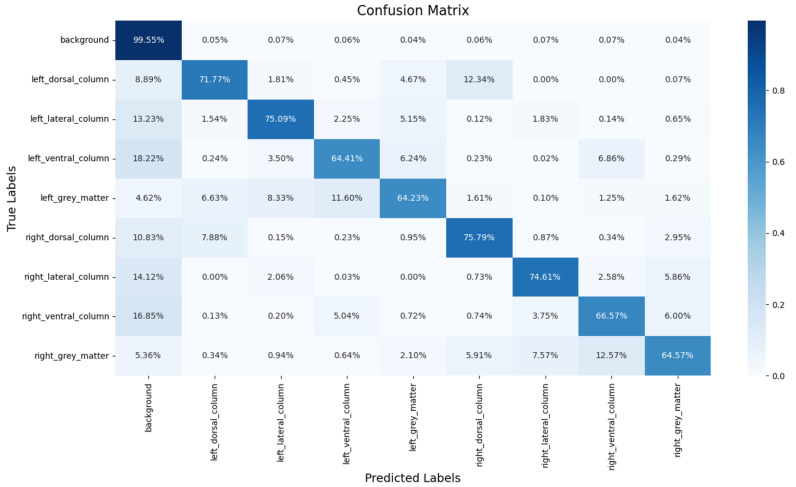
The segmentation results predicted on the sample DTI slice.

Figure 7 shows the confusion matrix of the proposed segmentation model, and the prediction ratios of each class are demonstrated. It is obvious that the most common misclassifications in the ROIs occurred between the ROIs and the background. On the other hand, misclassifications were also found between symmetric regions. For example, the left_dorsal_column (12.34%) and right_dorsal_column (7.88%) exhibited mutual misclassifications, with the former being confused with the latter by 11.60% and vice versa by 12.57%. Similarly, confusions between the left_ventral_column (6.86%) and right_ventral_column (5.04%) were also observed.

The detailed performance of the selected segmentation model is summarized in Table 3, which reports the precision, recall, and corresponding Dice score. The model demonstrated a higher recall compared to precision, which may be attributed to the penalty weight settings discussed earlier. These settings encouraged the segmentation model to prioritize capturing as many correct ROI pixels as possible. As a result, the model’s emphasis on recall may have led to the inclusion of background pixels near the boundaries of each ROI within the image. Moreover, the enhanced recall aligns with cost–utility considerations in the medical prediction task. In medical image segmentation, a higher fault tolerance rate is often acceptable when segmentation results include more background pixels. Conversely, incorrectly excluding non-background pixels can pose a greater risk and may adversely impact the diagnostic decisions made by clinicians. Therefore, the model’s focus on recall is designed to minimize missed detections, which is critical for ensuring reliable and accurate diagnostic outcomes.

### 3.2. DTI Specific Feature

In addition to the evaluation metrics commonly used for general segmentation tasks, FA-value-based assessment indices are also presented. Table 4 provides a detailed summary of the Mean FA error results for each ROI by comparing the labeled results with the predicted segmentations. Specifically, the column ‘Label mean FA’ represents the FA values calculated based on expert-labeled ROI voxels, while the column ‘Measured mean FA’ represents the FA values calculated based on the model-predicted ROI voxels. In the majority of ROIs, the mean FA error between the expert-labeled and model-predicted segmentations was less than 10%. This observation indicates that the segmentation model not only performed well for standard evaluation metrics but also achieved a high degree of accuracy in preserving FA value consistency. Such consistency is critical for clinical applications, where precise FA measurements are essential for downstream diagnostic and therapeutic decision-making.

### 3.3. DTI Segmentation Visualization

**Figure 8:**
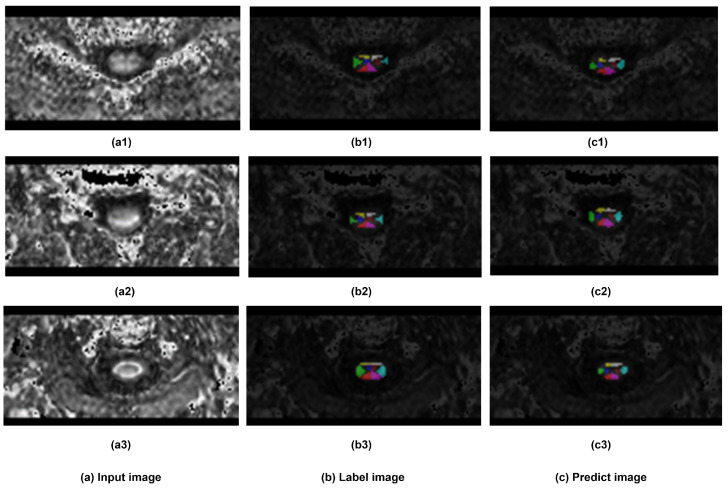
The Visualization of segmentation results. Three samples has been demonstrated. (**a**) sample input DTI slice, (**b**) visualization of labeled ground truth, and (**c**) visualization of segmentation prediction.

In terms of segmentation visualization, Figure 8 illustrates the actual segmentation outcomes generated by the proposed method, in this figure, each class (except the background) has been assigned a unique color. By comparing the visualization of the predicted segmentation results with the labeled ground truth image, the visual result clearly shows that the predicted segments were closely related to the corresponding labeled locations across each spinal cord segment.

### 3.4. Model Complexity Evaluation

Regarding the model complexity, Table 5 presents the Floating-point Operations Per Second (FLOPs) and parameter counts for all compared models. The results indicate that the proposed SCS-Net had the lowest FLOPs, while maintaining a moderate number of parameters. This reveals the lightweight design of SCS-Net, particularly in terms of computational efficiency. In contrast, models like Trans-Unet demonstrated significantly higher FLOPs and parameter counts, reflecting their heavier design. Similarly, Unet-VG19 and Unet-N, despite having lower parameter counts than SCS-Net, exhibited substantially higher FLOPs, suggesting less efficient internal designs.

## 4. Discussion

This research endeavored to establish an easily accessible auto-segmentation method or pipeline to support medical staff in clinical decision-making. To achieve this objective, this work contributes to three critical areas: The effective analysis of different inputs for DTI segmentation, performance improvements of the proposed SCS-Net model, and method rationality within DTI radiology. Additionally, the functional capacity of the segmentation pipeline was also discussed. This section will discuss how these aspects have been addressed.

First, we analyzed the contribution of different candidate images to segmentation performance. The experiment was designed to evaluate the performance of the same segmentation methods with different input sources. As depicted in Figure 5, the Dice coefficient results for these settings are presented. It was observed that models using B0 images as input yielded better results compared to those using FA images. This enhanced performance with B0 images is likely attributable to their clearer anatomical boundary delineation. This explanation was further supported by feedback from experts involved in the manual annotation process. On the other hand, the reason for the modest segmentation performance when the model used FA features is under investigated. One possible explanation is the changing of the FA value caused by neural damage confused the model in predicting the correct segmentation, as deep learning models are vulnerable during prediction when the accepted feature values are changed. However, it must be clarified that, based on the current state of knowledge, no quantitative relationship has been established to define a specific range of FA values where model performance degrades, and the mechanism of the effect of FA features on segmentation performance remains unclear.

In terms of the motivation for applying B0 images for DTI segmentation, as the comparison experiment of input images verified the effectiveness of B0 images, there is an intuitive strategy for improving DTI segmentation by directly projecting the ROI prediction from B0 image onto DTI images. The registration of DTI images originally referenced the B0 images, naturally exhibiting spatial and semantic consistency in ROI localization, making the projection strategy both intuitive and effective.

Moreover, the motivation for using B0 images rather than other anatomical images (such as T2-weighted MRI) for assisting DTI segmentation was due to the potential advantages in promoting the clinical adoption of DTI. Because the collection of B0 images is a inherent step in DTI registration, directly leveraging B0 images makes the DTI segmentation/analysis process independent of the involvement of additional image modalities, thus enhancing its accessibility in clinical applications. Additionally, this approach eliminates the costs and complexities associated with the collection and registration of cross-modality images, further supporting its feasibility for widespread clinical use.

Second, based on the B0-image-based segmentation-projection strategy, we further analyzed the performance improvement of the proposed model when using B0 images, which represents a key consideration in the model structure design. Figure 5 demonstrates that, when employing the same B0 image input, Unet-N outperformed Unet-RS50. This superior performance of the Unet-N encoder can likely be attributed to its relatively simplified VGG-based structure, which was better suited for the relatively limited size of the training dataset. Furthermore, Figure 6 compares the performance of Unet-N, Unet-VG19, SCS-Net, and Trans-Unet under B0 input conditions. The results indicate that the proposed SCS-Net achieved the best overall performance. Considering the low FLOPs of SCS-Net demonstrated in Table 5, the improved results suggest the design of SCS-Net gives it reduced model-complexity-dependent data requirements, which may effectively mitigate the underfitting in model training. Additionally, this design outperformed models based on the VGG backbone. While ResNet-based models generally outperform VGG-based models in various computer vision tasks due to their greater learning capacity [31,32], the most plausible explanation for the superior performance of SCS-Net is that its residual-based feature extraction structure enabled a substantial improvement once the issue of underfitting had been addressed. In addition, the performance of SCS-Net significantly surpassed Trans-Unet, indicating that Trans-Unet could not fit the current dataset. Considering that Transformer-based models have produced state-of-the-art performance on many computer vision datasets, we infer that the insufficient sample size was the main constraint on such performance. However, we believe that Transformer-based models hold significant potential for future research in DTI segmentation, especially once the training sample size has been expanded.

To further evaluate the misclassification of SCS-Net, a confusion matrix is provided. Notably, most non-background voxels (spinal cord voxels) were correctly detected by SCS-Net, aligning with the results presented in Table 3. The primary misclassifications arose from two aspects: (1) misclassifications between ROIs and the background, and (2) misclassifications between symmetrical ROIs. For the misclassifications between ROIs and the background, this issue likely reflects the inherent challenge of fuzzy boundary delineation in these regions. A voxel in DTI represents a fixed spatial resolution of the cervical disc (1 × 1.26 × 7 mm^3^ in this experiment), which does not correspond to the natural boundary between the ROI and the background. Consequently, boundary pixels are prone to misclassification, as they may contain both ROI and background structures. Moreover, misclassifications were also observed between symmetrical ROIs, such as the eft_dorsal_column (12.34%) and right_dorsal_column (7.88%). This finding highlights a direction for further improvement, especially in addressing symmetrical voxel/pixel localization in general semantic segmentation tasks or applying an instance segmentation method [33,34].

Thirdly, beyond general segmentation evaluation methods, another focus is on the radiological rationality inherent in DTI itself. As previously mentioned, DTI-assisted diagnosis requires FA value analysis, implying that a reliable segmentation prediction should have similar FA distributions as the ground truth labels. Table 4 compares the predicted segmentation results of SCS-Net against the ground truth.

First, the mean FA value in the background was found to be significantly different from that in the labeled ROIs. This result demonstrates that the predicted segments were not random and were effectively separated from the background, supporting the validity of the segmentation outputs. Second, the majority of segmentation classes exhibited a mean FA error of less than 10%, which reinforces the radiological rationality of the segmentation model. Notably, although the quantitative relation of mean FA error in affecting clinically decision-making remains unclear, based on the current reported result, the FA value measured at the affected cervical disc in CSM patients was significantly lower (more than 20%) than the unaffected cervical disc in CSM patients and/or the normal discs in controls [35,36]. Therefore, the current mean FA error of the proposed model (10%) can be inferred to have a limited impact on downstream diagnostic or prognostic processes.

Additionally, the similar spital distributions observed by comparing the visualized segmentation results with the original image in Figure 7 also suggests the consistent performance of the proposed SCS-Net.

As for the limitations of this work, the discussions above suggest that the segmentation model is capable of producing highly reliable results. However, potential limitations of this work include the following: Firstly, due to resource constraints, the data were only collected from single center, and the segmentation model’s generalization ability has thus not been fully investigated. Second, due to data scarcity, this research has not fully explored the performance of other advanced segmentation technologies, such as the prevalent Attention Mechanism and Transformer-based models. Thirdly, the utility of diffusive features for segmentation performance improvements requires further investigation, and FA value (or other diffusive features)-assisted analysis has not yet been confirmed as an effective method for improving DTI segmentation in model training, which limited the model’s ability to incorporate DTI radiological rationality during the training process.

## 5. Conclusions

In conclusion, this research proposed a deep-learning-based segmentation model for CSM DTI segmentation, the performance of the proposed model in both FA-value-based analysis and general segmentation evaluation proved its rationality in both clinical radiology and computer vision. Meanwhile, the encapsulated segmentation pipeline can enable clinicians to easily access the automated segmentation outputs of DTI slices through simple command operations. Thus, this work could be easily applied for medical staff to acquire preliminary segmentation results. We believe this work can support DTI-based clinical decision-making and further related research.

## Figures and Tables

**Table 1:** Parameters of acquired DTI.

Parameter Item	Choice	Uint
Device and Version	Philips 3T Achieva scanner	N/A
field of view	80 × 80	mm^2^
thickness of slices	7	mm
gap between slices	2.2	mm
fold-over direction	anteroposterior	N/A
reconstruction resolution	0.63 × 0.63 × 7	mm^3^
voxel resolution	1.0 × 1.26 × 7	mm^3^
TE/TR	60/5	ms/heartbeats

TE (Echo Time), TR (Repetition Time).

**Table 2:** The Deep learning training parameter settings in the experiment.

Parameter Item	Choice
Optimizer	Adam
Learning rate	1 × 10^−10^
Momentum	0.9
Batch size	16
Training epoch	500
Learning rate	1 × 10^−10^
input	FA/B0
Encoder structure	Vgg-16/Vgg-19/Resnet-50/Trans-Unet/SCS-Net

**Table 3:** General segmentation evaluation index results for each ROI in the DTI slice.

Segmentation Part	Dice	Precision	Recall
Left dorsal	0.5795±0.0335	0.4868±0.0364	0.7177±0.0395
Left lateral	0.5905±0.0336	0.4883±0.0387	0.7509±0.0476
Left ventral	0.4695±0.0319	0.3706±0.0314	0.6441±0.0498
Left gray matter	0.4907±0.0312	0.3974±0.0297	0.6423±0.0381
Right dorsal	0.6020±0.0292	0.4998±0.0272	0.7579±0.0430
Right lateral	0.5810±0.0258	0.4769±0.0267	0.7461±0.0476
Right ventral	0.4835±0.0363	0.3805±0.0328	0.6657±0.0565
Right gray matter	0.4938±0.0350	0.4002±0.0306	0.6457±0.0475
Mean	0.5363±0.0321	0.4376±0.0317	0.6963±0.0462

**Table 4:** The results of FA value error for each ROI in the DTI slice.

Segmentation Part	Label Mean FA	Measured Mean FA	Mean FA Error
Left dorsal	0.5950±0.0132	0.5635±0.0214	5.32%±2.15%
Left lateral	0.6033±0.0100	0.5421±0.0119	10.14%±1.26%
Left ventral	0.5674±0.0139	0.5255±0.0139	7.37%±2.03%
Left gray matter	0.5442±0.0072	0.5751±0.0084	5.70%±2.03%
Right dorsal	0.5894±0.0112	0.5624±0.0194	4.60%±1.95%
Right lateral	0.5418±0.0118	0.4897±0.0208	9.60%±3.76%
Right ventral	0.5833±0.0103	0.5324±0.0152	8.74%±1.60%
Right gray matter	0.5429±0.0103	0.5753±0.0109	6.27%±2.11%
Mean	0.5709±0.0086	0.5753±0.0138	6.48%±2.11%
Background	0.1812±0.0019	0.1802±0.0020	0.54%±0.15%

**Table 5:** Model complexity index.

Model Name	FLOPs	Parameters Count
SCS-Net	82.551G	34.365M
Unet-VG19	247.395G	30.2005M
Unet-RS50	91.739G	43.927M
Trans-Unet	467.404G	91.524M
Unet-N	225.651G	24.891M

## Data Availability

The original contributions presented in this study are included in the article. Further inquiries can be directed to the corresponding author.

## Appendix A

The training loss of SCS-Net was monitored across 500 epochs, with the best model saved based on the lowest validation loss. It was observed that the training loss decreased significantly during the early stages of the training process and continued to decline gradually throughout the remaining epochs.

Considering the error analysis discussed in the Discussion section, the significant loss reduction in the early stages can be attributed to the predominant ratio of background pixels, which were relatively easy to classify correctly. As the training progressed, the model focused on learning to correctly classify the more challenging pixels within the ROIs, resulting in a slower but consistent reduction in loss over time.
